# Incidence of and risk factors for postintubation hypotension in critically ill patients with COVID-19

**DOI:** 10.5935/0103-507X.20220007-en

**Published:** 2022

**Authors:** Bişar Ergün, Begüm Ergan, Mehmet Nuri Yakar, Murat Küçük, Murat Özçelik, Erdem Yaka, Ali Necati Gökmen

**Affiliations:** 1 Department of Internal Medicine and Critical Care, Faculty of Medicine, Dokuz Eylül University - Izmir, Turkey.; 2 Department of Pulmonary and Critical Care, Faculty of Medicine, Dokuz Eylül University - Izmir, Turkey.; 3 Department of Anesthesiology and Critical Care, Faculty of Medicine, Dokuz Eylül University - Izmir, Turkey.; 4 Neurology and Critical Care, Faculty of Medicine, Dokuz Eylül University - Izmir, Turkey.

**Keywords:** COVID-19, Coronavirus infections, SARS-CoV-2, Hospital mortality, Hypotension, Critical illness, Intubation, intratracheal, Risk factors, Intensive care units

## Abstract

**Objective:**

To evaluate the incidence of risk factors for postintubation hypotension in critically ill patients with COVID-19.

**Methods:**

We conducted a retrospective study of 141 patients with COVID-19 who were intubated in the intensive care unit. Postintubation hypotension was defined as the need for any vasopressor dose at any time within the 60 minutes following intubation. Patients with intubation-related cardiac arrest and hypotension before intubation were excluded from the study.

**Results:**

Of the 141 included patients, 53 patients (37.5%) had postintubation hypotension, and 43.6% of the patients (n = 17) were female. The median age of the postintubation hypotension group was 75.0 (interquartile range: 67.0 - 84.0). In the multivariate analysis, shock index ≥ 0.90 (OR = 7.76; 95%CI 3.14 - 19.21; p < 0.001), albumin levels < 2.92g/dL (OR = 3.65; 95%CI 1.49 - 8.96; p = 0.005), and procalcitonin levels (OR = 1.07, 95%CI 1.01 - 1.15; p = 0.045) were independent risk factors for postintubation hypotension. Hospital mortality was similar in patients with postintubation hypotension and patients without postintubation hypotension (92.5% *versus* 85.2%; p = 0.29).

**Conclusion:**

The incidence of postintubation hypotension was 37.5% in critically ill COVID-19 patients. A shock index ≥ 0.90 and albumin levels < 2.92g/dL were independently associated with postintubation hypotension. Furthermore, a shock index ≥ 0.90 may be a practical tool to predict the increased risk of postintubation hypotension in bedside scenarios before endotracheal intubation. In this study, postintubation hypotension was not associated with increased hospital mortality in COVID-19 patients.

## INTRODUCTION

Endotracheal intubation (ETI) is a common procedure that is performed in critically ill patients.^(1)^ Complications such as hypoxemia, aspiration, hypotension, and cardiac arrest are common during ETI procedures in critically ill patients.^(2)^ Moreover, intubation-related cardiac arrest has been detected with an incidence of 2.7% in a study conducted in an intensive care unit (ICU).^(3)^ Postintubation hypotension (PIH), which is one of the most serious complications, has been reported with an incidence of 20 - 52% in ICU patients.^(4-8)^ Postintubation hypotension is associated with increased ICU and in-hospital mortality,^(4-6,8)^ as well as increased length of stay.^(8)^

There is no consensus on the definition of PIH in the literature. The most accepted criteria include a decrease in systolic blood pressure (SBP) or mean arterial pressure (MAP), the initiation or increase in any vasopressor (bolus or infusion), or fluid administration (crystalloid and/or colloid) of ≥ 30mL/kg at any time within 30 minutes or 60 minutes postintubation.^(4,7,8)^ Additionally, it has been reported that vasoactive administration at 60 minutes after ETI has a higher association with in-hospital and 90-day mortality.^(9)^

Coronavirus disease 2019 (COVID-19) is a pneumonia outbreak caused by severe acute respiratory syndrome coronavirus 2 (SARS-CoV-2).^(10)^ Invasive mechanical ventilation (IMV) is the cornerstone of treatment in patients with severe COVID-19.^(11)^ In a study evaluating the outcomes of 2,634 patients who were hospitalized with COVID-19, 12.2% of the patients received IMV.^(12)^ In a meta-analysis including 57,420 adult patients with COVID-19 who received IMV, the overall reported case fatality rate (CFR) was estimated to be 45% (95% confidence interval - 95%CI 39 - 52%).^(11)^ Due to the widespread use of IMV in severe COVID-19 pneumonia, intubation-related complications need to be considered, as they may have an impact on patient outcomes.

In a recent study performed on 202 COVID-19 patients who underwent ETI, four patients (2%) developed cardiac arrest, and hypotension (defined as an arterial pressure < 90/60mmHg) occurred in 22.3% (n = 45) of patients after ETI.^(13)^ In another study involving 53 COVID-19 patients, there was no cardiac arrest, but 21% (n = 11) of patients developed hypotension (defined as SBP < 90 mmHg) after ETI.^(14)^

To our knowledge, there are no studies that have specifically analyzed risk factors for PIH in critically ill patients with COVID-19. In this study, the primary aim was to determine the potential risk factors for PIH in severe COVID-19 patients. Secondary aims were to determine the incidence of PIH and its impact on the length of stay in the hospital or ICU, as well as hospital or ICU mortality.

## METHODS

After approval from the local ethics committee (with an approved date of 2021/02/01 and an approval number of 2021/03-08) and the Turkish Ministry of Health, the retrospective cohort study was conducted in adult ICUs of our center. All of the consecutive patients (age ≥ 18 years) who were diagnosed with COVID-19 infections and who underwent ETI between March 2020 and February 2021 were included in the study. SARS-CoV-2 infection was confirmed by using reverse transcriptase polymerase chain reaction (RT-PCR) testing on respiratory samples and/or clinical characteristics, as well as laboratory and computed tomography findings. The exclusion criteria were as follows: ETI before ICU admission; ETI due to cardiac arrest; cardiac arrest during or immediately after ETI; any SBP less than 90mmHg and any MAP less than 65mmHg within 60 minutes before ETI; vasopressor support within 60 minutes before ETI; unavailable blood pressure and medication data; reintubations in the same patient; more than two attempts for ETI; and uncontrolled cardiac arrhythmias before ETI.

The demographic data (age, sex, body mass index, smoking history, and comorbidities), Charlson Comorbidity Index (CCI), Acute Physiology and Chronic Health Evaluation (APACHE) II, and Sequential Organ Failure Assessment (SOFA) scores were recorded. Disease characteristics for COVID-19, including the date of symptom onset, RT-PCR results, and radiological tests (either chest X-ray or computerized tomography of the chest), were collected. Laboratory data, including hemogram, albumin, creatinine, D-dimer, B-type natriuretic peptide, and procalcitonin, were recorded for at least 24 hours before ETI. Arterial blood gas parameters were recorded as parameters immediately before ETI.

Major events during the ICU stay (presence of septic shock, ICU-acquired infections, mechanical ventilation support, acute kidney injury - AKI, and renal replacement therapy) were recorded. Lengths of ICU stay and hospital stay, as well as mortality, were recorded.

### Intubation protocol in the intensive care unit setting

The ETI decision was made according to the clinical severity of acute respiratory failure and was performed according to the COVID-19 guidelines of the Turkish Ministry of Health, which recommends rapid intubation with an expert team.^(15)^ Intubations were performed in the negative pressure room of the ICU (when unavailable) in a standard ICU room. Endotracheal intubation was performed according to the sequential intubation protocol as much as possible. All of the intubations were implemented through the use of a video laryngoscope. All of the service personnel in charge of the intubation paid maximum attention to personal protective equipment.

Intubation clinical data included preintubation heart rate, SBP, MAP, oxygen saturation with pulse oximetry, the need for noninvasive ventilation (NIV) or highflow nasal cannula (HFNC) oxygen therapy 6 hours before intubation, medications used for induction, the specialties of the physicians (anesthesia or intensive care), and (if present) the time of cardiac arrest. Drug and fluid replacements that were administered within 24 hours before ETI were recorded. Moreover, total fluid balance was recorded within 48 hours prior to ETI. Ventilator settings and vasopressor use were recorded within 60 minutes of ETI.

### Definitions

In this study, PIH was defined as the need for any vasopressor dose (norepinephrine) at any time within the 60 minutes following ETI, as described in the literature.^(8,9)^ Baseline blood pressure and heart rate were defined by using values that were recorded immediately before the administrations of hypnotic agents and neuromuscular blocking agents for ETI. The shock index (SI) was defined as the heart rate divided by SBP, and the modified shock index (MSI) was defined as the heart rate divided by the MAP that was measured immediately before the administration of hypnotic agents and neuromuscular blocking agents for ETI.^(16,17)^ Patients with a SI value ≥ 0.90 preETI were recorded because this value was significantly associated with PIH in the literature.^(16)^ To assess the effect of albumin levels on PIH, patients were divided into two groups according to the median albumin level (2.92g/dL). Acute kidney injury was identified according to the Kidney Disease: Improving Global Outcomes (KDIGO) definition.^(18)^

### Statistical analysis

The primary outcome of the study was to determine the potential risk factors for PIH in severe COVID-19 patients. Secondary outcomes were to determine the incidence of PIH and the impact of PIH on the lengths of stay in the hospital or ICU, as well as hospital or ICU mortality. All of the categorical variables are expressed as numbers and percentages, and continuous variables are expressed as medians and interquartile ranges. Categorical variables between groups were compared with the Chi-squared test or Fisher’s exact test, and continuous variables were compared with the Mann-Whitney *U* test. The independent risk factors for PIH were assessed via a multivariate logistic regression analysis. To build the model, a purposeful selection method was used to select a subset of the covariates that were considered clinically important, with adjustments for confounders and statistical significance. An adjusted odds ratio (OR) and a 95%CI were reported for each independent factor. A two-tailed p-value of 0.05 was considered to be statistically significant. Statistical analyses were performed by using Statistical Package for the Social Sciences (SPSS) Version 24 (IBM Corp., Armonk, N.Y., USA)

## RESULTS

### General characteristics

**Figure 1:**
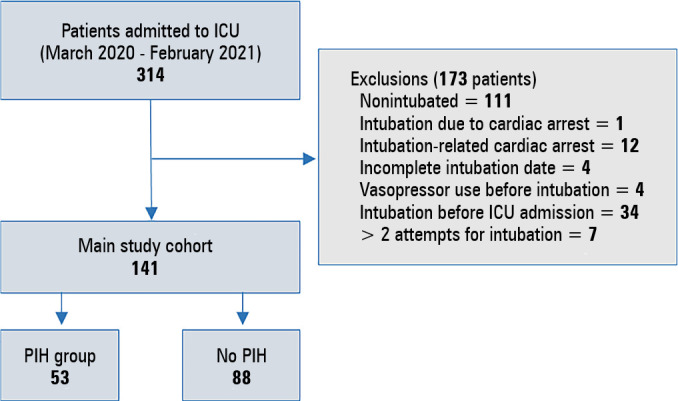
Study population. ICU - intensive care unit; PIH - postintubation hypotension.

During the study period, 141 of 314 patients admitted to the ICU with COVID-19 infection were included in the study. Of them, 53 patients (37.6%) had PIH (Figure 1).

The median age of all of the patients in this study was 72.0 (64.5 - 81.0) years (Table 1). A total of 72.3% (n = 102) of the patients were male, and 27.7% (n = 39) of the patients were female. Patients had complicated preexisting comorbidities, with a median CCI of 5.0 (3.0 - 7.0).

**Table 1:** Demographic and clinical characteristics of patients with and without postintubation hypotension (univariate analysis)

Characteristics	All cases	PIH	No PIH	p value
	**(n = 141)**	**(n = 53)**	**(n = 88)**	
Age (years)	72.0 (64.5 - 81.0)	75.0 (67.0 - 84.0)	71.0 (62.0 - 80.0)	0.048
Sex Female	39 (27.7)	17 (43.6)	22 (56.4)	0.44
Male	102 (72.3)	36 (35.3)	66 (29.4)	
Body mass index (kg/m^2^)	26.1 (23.6 - 29.3)	26.0 (23.5 - 29.9)	26.2 (24.0 - 29.1)	0.68
Smoking history	34 (24.1)	9 (17.0)	25 (28.4)	0.16
RT-PCR positivity	133 (94.3)	49 (92.5)	84 (95.5)	0.47
Comorbidities Chronic kidney disease	32 (22.7)	18 (34.0)	14 (15.9)	0.02
Hypertension	103 (73.0)	41 (77.4)	62 (70.5)	0.44
Diabetes mellitus	60 (42.6)	18 (34.0)	42 (47.7)	0.12
Coronary artery disease	39 (27.7)	16 (30.2)	23 (26.1)	0.70
Congestive heart failure	21 (14.9)	11 (20.8)	10 (11.4)	0.15
Atrial fibrillation	10 (7.1)	4 (7.5)	6 (6.8)	1.00
Neurological disease*	29 (20.6)	15 (28.3)	14 (15.9)	0.09
COPD	17 (12.1)	8 (15.1)	9 (10.2)	0.43
Hyperlipidemia	16 (11.3)	6 (11.3)	10 (11.4)	1.00
Malignancy†	13 (9.2)	2 (3.8)	11 (12.5)	0.13
APACHE II	23.0 (14.0 - 28.0)	25.0 (16.0 - 28.5)	21.5 (13.3 - 27.0)	0.027
SOFA ‡	6.0 (4.0 - 7.0)	6.0 (5.0 - 9.5)	5.0 (4.0 - 7.0)	0.005
CCI	5.0 (3.0 - 7.0)	6.0 (4.5 - 7.5)	5.0 (3.0 - 6.0)	0.012
Laboratory data within 24 hours before ETI BUN (mg/dL)	31.0 (23.0 - 52.0)	41.0 (26.5 - 57.3)	28.2 (21.0 - 45.0)	0.009
Creatinine (mg/dL)	0.99 (0.76 - 1.62)	1.11 (0.75 - 1.87)	0.98 (0.76 - 1.37)	0.23
Albumin (g/dL)	2.92 (2.58 - 3.16)	2.67 (2.47 - 3.13)	2.97 (2.65 - 3.28)	0.008
Patients with albumin < 2.92	70 (49.6)	34 (64.2)	36 (40.9)	0.009
Total bilirubin (mg/dL)	0.95 (0.65 - 1.22)	1.00 (0.76 - 1.48)	0.82 (0.60 - 1.12)	0.01
Procalcitonin (ng/mL)	0.52 (0.19 - 1.97)	0.96 (0.29 - 3.46)	0.34 (0.15 - 1.12)	0.002
CRP (mg/dL)	160 (92 - 243)	187 (102 - 235)	148 (85 - 247)	0.57
D-dimer (µg/mL)	2.00 (1.20 - 3.95)	2.90 (1.60 - 6.90)	1.65 (1.00 - 3.55)	0.01
Hs-Troponin I (ng/L)	30.3 (11.0 - 120.3)	60.0 (16.9 - 284.5)	26.2 (9.9 - 101.0)	0.06
BNP (plasma) (pg/mL)	102 (45 - 298)	113 (44 - 337)	100 (45 - 291)	0.70
ALT (U/L)	36.0 (23.0 - 62.0)	36.0 (21.0 - 63.5)	35.5 (23.2 - 62.5)	0.61
AST (U/L)	57.0 (39.5 - 89.5)	53.0 (38.5 - 117.5)	57.5 (39.2 - 82.2)	0.88
LDH (U/L)	584 (456 - 722)	586 (485 - 803)	583 (433 - 693)	0.21
Ferritin (ng/mL)	633 (375 - 1062)	654 (328 - 1222)	613 (380 - 879)	0.45
WBC, x 10^3^/µL	12.1 (8.5 - 15.2)	11.8 (9.0 - 16.1)	12.2 (8.2 - 15.1)	0.48
Neutrophil, x 10^3^/µL	10.4 (7.2 - 14.4)	10.6 (7.2 - 14.6)	10.1 (7.1 - 14.1)	0.54
Lymphocyte, x 10^3^/µL	0.5 (0.4 - 0.9)	0.5 (0.4 - 0.9)	0.5 (0.3 - 0.9)	0.84
Lymphocyte percentages (%)	5.1 (2.7 - 8.4)	4.7 (2.8 - 8.0)	5.6 (2.6 - 8.5)	0.55
Hemoglobin (g/dL)	12.2 (10.5 - 13.6)	11.8 (10.4 - 13.8)	12.3 (10.6 - 13.5)	0.64
Platelet, x 10^3^/µL	266 (192 - 349)	255 (184 - 357)	269 (198 - 346)	0.96
Arterial blood gas analysis just before ETI or within 6 hours before ETI pH	7.40 (7.30 - 7.47)	7.37 (7.29 - 7.46)	7.40(7.32 - 7.48)	0.15
PaO2 (mmHg)	58.0 (50.0 - 65.0)	54.0 (48.5 - 61.5)	59.5 (50.3 - 68.0)	0.06
PaCO2 (mmHg)	36.0 (30.0 - 45.0)	36.0 (29.5 - 44.0)	35.0 (30.0 - 45.8)	0.82
HCO3 (mmol/L)	22.2 (19.4 - 25.0)	22.0 (18.4 - 25.0)	23.0 (20.9 - 25.2)	0.052
Lactate (mmol/L)	2.10 (1.45 - 3.00)	2.50 (1.70 - 4.05)	2.00 (1.40 - 2.60)	0.009
SO2 (%)	83.0 (88.5 - 92.0)	88.0 (81.0 - 91.0)	89.5 (84.2 - 93.0)	0.04
PaO2/FiO2	100 (87 - 117)	95 (83 - 110)	102 (88 - 120)	0.055

PIH - postintubation hypotension; RT-PCR - reverse transcription-polymerase chain reaction; COPD - chronic obstructive pulmonary disease; APACHE II - Acute Physiology and Chronic Health Evaluation II; SOFA - Sequential Organ Failure Assessment; CCI - Charlson Comorbidity Index; ETI - endotracheal intubation; BUN - blood urea nitrogen; CRP - C-reactive protein; Hs - high-sensitivity; BNP - B-type natriuretic peptide; ALT - alanine transaminase; AST - aspartate transaminase; LDH - lactate dehydrogenase; WBC - white blood cell; PaO2 - partial pressure of arterial oxygen; PaCO2 - partial pressure of arterial carbon dioxide; HCO3 - bicarbonate; SO2 - arterial oxygen saturation; FiO2 - fraction of inspired oxygen.

* History of cerebrovascular disease or dementia; † includes hematological and solid organ malignancies; ‡ calculated on the day of intensive care unit admission. Results are expressed as n (%) or medians (interquartile range).

The incidence of PIH in women was not different from that in men (43.6% and 35.3%, respectively; p = 0.44). Patients with PIH were significantly older (75.0 [67.0 - 84.0] years *versus* 71.0 [62.0 - 80.0] years; p = 0.048) and had a higher prevalence of chronic kidney disease (34.0% *versus* 15.9%; p = 0.02) than patients without PIH. In patients with PIH, the median APACHE II score was higher than that in patients without PIH (25.0 [16.0 - 28.5] *versus* 21.5 [13.3 - 27.0]; p = 0.027). Moreover, patients with PIH had a higher median SOFA than patients without PIH (6.0 [5.0 - 9.5] *versus* 5.0 [4.0 - 7.0]; p = 0.005). The CCI median score was higher in patients with PIH than in patients without PIH (6.0 [4.5 - 7.5] *versus* 5.0 [3.0 - 6.0]; p = 0.012).

### Laboratory findings

In patients with PIH, median albumin levels were lower than in patients without PIH (2.67 [2.47 - 3.13] g/dL *versus* 2.97 [2.65 - 3.28] g/dL; p = 0.008). The rate of patients with albumin < 2.92g/dL was higher in the PIH group than in the non-PIH group (64.2% *versus* 40.9%; p = 0.009). In patients with PIH, median lactate levels were higher than in patients without PIH (2.50 [1.70 - 4.05] mmol/L *versus* 2.00 [1.40 - 2.60] mmol/L; p = 0.009). In patients with PIH, median procalcitonin levels were higher than in patients without PIH (0.96 [0.29 - 3.46] ng/mL *versus* 0.34 [0.15 - 1.12] ng/mL; p = 0.002). In patients with PIH blood urea nitrogen levels, total bilirubin levels and D-dimer levels were also higher than those levels in patients without PIH.

### Clinical characteristics before, during, and after endotracheal intubation

In patients without PIH, fentanyl use was more frequent than in the group with PIH (94.3% *versus* 83.0%; p = 0.04; Table 2). The other types of drugs and their doses that were used during ETI were not significantly different in either group. There was no difference between the two groups in terms of antihypertensive, antiarrhythmic, or sedative-hypnotic drugs that were used within 24 hours before ETI.

**Table 2:** Variables obtained before, during, and immediately after endotracheal intubation

Variables	All cases	PIH	No PIH	p value
	**(n = 141)**	**(n = 53)**	**(n = 88)**	
ETI medications Midazolam	131 (92.9)	48 (90.6)	83 (94.3)	0.50
Midazolam dose (mg/kg)	0.04 (0.03 - 0.06)	0.03 (0.03 - 0.06)	0.04 (0.03 - 0.06)	0.30
Propofol	4 (2.8)	3 (5.7)	1 (1.1)	0.15
Ketamine	6 (4.3)	2 (3.8)	4 (4.5)	1.00
Fentanyl	127 (90.1)	44 (83.0)	83 (94.3)	0.04
Fentanyl dose (mcg/kg)	1.60 (1.30 - 2.00)	1.60 (1.15 - 2.15)	1.60 (1.40 - 2.00)	1.00
Rocuronium	141 (100)	53 (100)	88 (100)	N/A
Rocuronium dose (mg/kg)	0.80 (0.70 - 1.10)	0.80 (0.70 - 1.05)	0.80 (0.70 - 1.10)	0.75
ETI events Difficult airway (two attempts)	7 (5.0)	4(7.5)	3 (3.4)	0.43
Medications administered within 24 hours before ETI Beta-blockers	39 (27.7)	19 (35.8)	20 (22.7)	0.12
Calcium channel blockers	33 (23.4)	14 (26.4)	19 21.6()	0.54
Diuretics	28 (19.9)	11 (20.8)	17 (19.3)	0.83
ACE-inhibitors and ARBs	26 (18.4)	8 (15.1)	18 (20.5)	0.50
Nitrates	5 (3.5)	2 (3.8)	3 (3.4)	1.00
Anti-arrhythmic agents	5 (3.5)	3 (5.7)	2 (2.3)	0.36
Alfa-blockers	2 (1.4)	1 (1.9)	1 (1.1)	1.00
Dexmedetomidine	23 (16.3)	8 (15.1)	15 (17.0)	0.82
Other sedative drugs	17 (12.1)	9 (17.0)	8 (9.1)	0.19
Fentanyl or other narcotics	3 (2.1)	2 (3.8)	1 (1.1)	0.56
Fluid management and oxygen support before ETI HFNC support pre-ETI (6 hours)	44 (31.2)	17 (32.1)	27 (30.7)	0.85
Noninvasive ventilation pre-ETI (6 hours)	37 (26.2)	14 (26.4)	23 (26.1)	1.00
Total crystalloid replacement (mL) pre-ETI (24 hours)	1,200 (750 - 2,145)	1,400 (700 - 2,363)	1,100 (806 - 1,803)	0.50
Negative fluid balance*	20 (14.2)	10 (18.9)	10 (11.4)	0.44
Pre-ETI hemodynamic and r espi r at or y assessment SBP (mmHg)	137 (112 - 151)	115 (100 - 140)	145 (125 - 158)	< 0.001
MAP (mmHg)	87 (72 - 100)	73 (68 - 91)	94 (80 - 106)	< 0.001
Heart rate (bpm)	103 (88 - 123)	114 (98 - 129)	100 (82 - 117)	0.004
SpO2 (%)	85 (78 - 90)	84 (95 - 91)	86 (78 - 90)	0.98
SI	0.78 (0.63 - 0.96)	0.93 (0.74 - 1.20)	0.68 (0.58 - 0.84)	< 0.001
SI ≥ 0.90	46 (32.6)	31 (58.5)	15 (17.0)	< 0.001
MSI	1.15 (0.97 - 1.43)	1.39 (1.13 - 1.76)	1.09 (0.92 - 1.28)	< 0.001
Hemodynamic, respiratory, and mechanical ventilation parameters assessment (60 min utes after ETI) SBP (mmHg)	121 (100 - 151)	99 (80 - 120)	140 (118 - 160)	< 0.001
MAP (mmHg)	83 (66 - 101)	63 (51 - 82)	91 (78 - 106)	< 0.001
SpO2 (%)	91 (84 - 95)	89 (80 - 94)	92 (86 - 95)	0.01
Tidal volume (mL/kg of PBW)	5.8 (5.0 - 7.0)	5.8 (4.7 - 7.4)	5.8 (5.2 - 6.8)	0.53
PEEP (mbar)	8.0 (6.0 - 15.0)	8.0 (7.0 - 19.0)	8.0 (6.0 - 12.0)	0.35
Sedative agents infusion	83 (58.9)	26 (49.1)	57 (64.8)	0.07
Analgesic agents infusion	93 (66.0)	30 (56.6)	63 (71.6)	0.09
Norepinephrine dose (mcg/kg/minute)	0.00 (0.00 - 0.04)	0.10 (0.03 - 0.38)	N/A	N/A
Vasopressors persisting 24 hours after ETI	41 (29)	41 (77.4)	N/A	N/A

PIH - postintubation hypotension; ETI - endotracheal intubation; N/A - not applicable; ACE - angiotensin converting enzymes; ARB - angiotensin receptor blockers; HFNC - high-flow nasal cannula; SBP - systolic blood pressure; MAP - mean arterial blood pressure; SpO2 - oxygen saturation; SI - shock index; MSI - modified shock index; PBW - predicted body weight; PEEP - positive end-expiratory pressure.

* n = 90, patients who were intubated within two days of admission were excluded. Results are expressed as n (%) or medians (interquartile range).

Patients with PIH had a lower SBP (115 [100 - 140] mmHg *versus* 145 [125 - 158] mmHg; p < 0.001), a lower MAP (73 [68 - 91] mmHg *versus* 94 [80 - 106] mmHg; p < 0.001), and a higher heart rate (114 [98 - 129] bpm *versus* 100 [82 - 117] bpm; p = 0.004) than patients without PIH immediately before ETI. Consistently, SI (0.93 [0.74 - 1.20] *versus* 0.68 [0.58 - 0.84]; p < 0.001), and MSI (1.39 [1.13 - 1.76] *versus* 1.09 [0.92 - 1.28]; p < 0.001) were higher in patients with PIH than in patients without PIH. In patients with SI values ≥ 0.90, PIH was more frequent than in patients with SI values lower than 0.90 (58.5% *versus* 17.0%; p < 0.001).

### Major events during intensive care unit stay and short-term outcomes

The overall AKI incidence in the entire cohort was 64.5% (n = 91). Acute kidney injury developed in the post-ETI period in 68 (48.2%) patients. Acute kidney injury rates were similar in PIH and no PIH groups (50.9% *versus* 46.6%; p = 0.73; Table 3). Hospital length of stay and ICU length of stay were similar in both groups. Hospital mortality was 87.9% (n = 124) in all of the study patients. Both ICU mortality (92.5% *versus* 84.1%; p = 0.20) and hospital mortality (92.5% *versus* 85.2%; p = 0.29) were higher in patients with PIH than in patients without PIH; however, the differences were not statistically significant.

**Table 3:** Events/therapies during intensive care unit stay and short-term outcomes in patients with and without postintubation hypotension (univariate analysis)

Events/Therapies	All cases	PIH	No PIH	p value
	**(n = 141)**	**(n = 53)**	**(n = 88)**	
Events/therapies of kidney diseases Chronic kidney disease	32 (22.7)	18 (34.0)	14 (15.9)	0.02
Dialysis-dependent	4 (2.8)	4 (7.5)	0 (0)	0.02
AKI, in the pre-ETI period	23 (16.3)	11 (20.8)	12 (13.6)	0.35
Newly RRT, in the pre-ETI period	2 (1.4)	1 (1.9)	1 (1.1)	1.00
AKI, in the post-ETI period	68 (48.2)	27 (50.9)	41 (46.6)	0.73
Newly RRT, in the post-ETI period	61 (43.3)	25 (47.2)	36 (40.9)	0.49
Nephrotoxic drugs	40 (28.4)	16 (30.2)	24 (27.3)	0.70
Time from ETI to onset of AKI, days	2.0 (1.0 - 4.0)	1.0 (1.0 - 3.0)	2.0 (1.0 - 4.0)	0.30
Events/therapies during the entire ICU stay Vasopressor requirement	122 (86.5)	53 (100)	69 (78.4)	< 0.001
Secondary infections	102 (72.3)	37 (69.8)	65 (73.9)	0.70
HFNC, before ETI	49 (34.8)	22 (41.5)	27 (30.7)	0.21
NIV, before ETI	29 (20.6)	12 (22.6)	17 (19.3)	0.67
Successful weaning	18 (12.8)	4 (7.5)	14 (15.9)	0.20
Tracheostomy	4 (2.8)	1 (1.9)	3 (3.4)	1.00
Treatment of COVID-19 Favipravir	133 (94.3)	48 (90.6)	85 (96.6)	0.15
LMWH	134 (95.0)	51 (96.2)	83 (94.3)	0.71
ASA	112 (79.4)	41 (77.4)	71 (80.7)	0.67
Dipyridamole	95 (67.4)	35 (66.0)	60 (68.2)	0.85
Corticosteroids	104 (73.8)	38 (71.7)	66 (75.0)	0.70
Pulse corticosteroid	55 (39.0)	21 (39.6)	34 (38.6)	1.00
Hydroxychloroquine	41 (29.1)	13 (24.5)	28 (31.8)	0.45
Tocilizumab	15 (10.6)	4 (7.5)	11 (12.5)	0.41
Time from symptom onset to ETI (days)	8.0 (4.0 - 11.0)	7.0 (4.0 - 11.5)	8.0 (5.0 - 11.0)	0.85
Time from hospital admission to ETI (days)	4.0 (1.0 - 7.0)	5.0 (2.0 - 9.0)	3.0 (1.0 - 6.0)	0.053
Time from ICU admission to ETI (days)	0.0 (0.0 - 3.0)	1.0 (0.0 - 3.5)	0.0 (0.0 - 2.0)	0.08
Hospital length of stay (days)*	26.0 (17.0 - 35.0)	33.5 (21.5 - 71.0)	24.0 (15.0 - 33.0)	0.20
ICU length of stay (days)*	18.0 (11.5 - 23.3)	20.0 (7.3 - 47.8)	17.5 (11.5 - 21.8)	0.80
ICU mortality	123 (87.2)	49 (92.5)	74 (84.1)	0.20
Hospital mortality	124 (87.9)	49 (92.5)	75 (85.2)	0.29

PIH - postintubation hypotension; AKI - acute kidney injury; ETI - endotracheal intubation; RRT - renal replacement therapy; ICU - intensive care unit; HFNC - high-flow nasal cannula; NIV - noninvasive ventilation; LMWH - low molecular weight heparin; ASA - acetylsalicylic acid.

* Length of stay in patients who survived. Results are expressed as n (%) or medians (interquartile range).

### Independent risk factors for postintubation hypotension

The multivariable analysis (Table 4) showed SI ≥ 0.90 (OR = 7.76, 95%CI 3.14 - 19.21; p < 0.001), albumin levels < 2.92g/dL (OR = 3.65, 95%CI 1.49 - 8.96; p = 0.005), and procalcitonin levels (OR = 1.07, 95%CI 1.01 - 1.15; p = 0.045) as factors independently associated with an increased risk of PIH.

**Table 4:** Logistic regression analysis for risk factors for postintubation hypotension

Risk factors	OR (95%CI)	p value
SI ≥ 0.90	7.76 (3.14 - 19.21)	< 0.001
Albumin < 2.92g/dL	3.65 (1.49 - 8.96)	0.005
Procalcitonin (ng/mL)	1.07 (1.01 - 1.15)	0.045
APACHE II	1.02 (0.96 - 1.08)	0.597
Charlson Comorbidity Index	1.07 (0.89 - 1.29)	0.484
Lactate (mmol/L)	1.23 (0.99 - 1.52)	0.060

OR - odds ratio; 95%CI - 95% confidence interval; SI - shock index; APACHE II - Acute Physiology and Chronic Health Evaluation II.

### Factors associated with hospital mortality

The median age was higher in patients who died than in patients who survived (74.0 [66.3 - 81.0] years *versus* 61.0 [55.0 - 71.5], years; p = 0.003; Table 5). The median CCI score was higher in patients who died than in patients who survived (6.0 [4.0 - 7.0] *versus* 4.0 [2.0 - 5.0]; p = 0.002).

**Table 5:** Statistically significant variables for hospital mortality (univariate analysis)

Characteristics	All cases (n = 141)	Dead group (n = 124)	Alive group (n = 17)	p value
Age (years)	72.0 (64.5 - 81.0)	74.0 (66.3 - 81.0)	61.0 (55.0 - 71.5)	0.003
Comorbidities Chronic kidney disease	32 (22.7)	32 (25.8)	0 (0)	0.013
SOFA	6.0 (4.0 - 7.0)	6.0 (4.0 - 8.0)	4.0 (4.0 - 6.0)	0.006
CCI	5.0 (3.0 - 7.0)	6.0 (4.0 - 7.0)	4.0 (2.0 - 5.0)	0.002
Laboratory data				
BUN (mg/dL)	31.0 (23.0 - 52.0)	36.0 (24.0 - 54.0)	24.0 (13.8 - 25.6)	< 0.001
Creatinine (mg/dL)	0.99 (0.76 - 1.62)	1.00 (0.78 - 1.69)	0.90 (0.61 - 1.02)	0.031
D-dimer (µg/mL)	2.00 (1.20 - 3.95)	2.15 (1.30 - 4.93)	1.30 (0.80 - 2.60)	0.018
WBC, x 103/µL	12.1 (8.5 - 15.2)	12.5 (9.3 - 15.3)	9.0 (7.0 - 11.1)	0.013
Neutrophil, x 103/µL	10.4 (7.2 - 14.4)	10.7 (7.5 - 14.5)	8.0 (6.1 - 9.7)	0.023
Arterial blood gas analysis HCO3 (mmol/L)	22.2 (19.4 - 25.0)	22.0 (19.0 - 25.0)	25.0 (22.0 - 26.0)	0.025
Pre-ETI hemodynamic and respiratory assessment Heart rate (bpm)	103 (88 - 123)	106 (89 - 125)	87 (77 - 104)	0.008
Hemodynamic, respiratory, and mechanical ventilation parameters assessment (60 minutes after ETI)				
SpO2 (%)	91 (84 - 95)	89 (83 - 94)	96 (94 - 98)	< 0.001
Events/therapies of kidney diseases				
Acute kidney injury, entire follow-up	91 (64.5)	85 (68.5)	6 (35.3)	0.013
Renal replacement therapy, entire follow-up	67 (47.5)	65 (52.4)	2 (11.8)	0.002
AKI, in the post-ETI period	68 (48.2)	64 (51.6)	4 (23.5)	0.038
Newly RRT, in the post-ETI period	61 (43.3)	59 (47.6)	2 (11.8)	0.007
Treatment of COVID-19 Pulse corticosteroid	55 (39.0)	53 (42.7)	2 (11.8)	0.016

SOFA - Sequential Organ Failure Assessment; CCI - Charlson Comorbidity Index; BUN - blood urea nitrogen; WBC - white blood cell count; HCO3 - bicarbonate; ETI - endotracheal intubation; SpO2 - oxygen saturation; AKI - acute kidney injury; RRT - renal replacement therapy. Results are expressed as n (%) or medians (interquartile range).

## DISCUSSION

This retrospective cohort study addressed the possible risk factors for PIH development in severe COVID-19 patients and demonstrated three important results. First, when using our definition for PIH, the incidence of PIH was 37.6% in critically ill COVID-19 patients. Second, SI ≥ 0.90, albumin levels < 2.92g/dL, and higher procalcitonin levels compared to lower procalcitonin levels were independently associated with PIH. Third, clinically important outcomes such as AKI development, length of stay, and mortality were similar in the PIH and non-PIH groups.

The incidence of PIH is 20 - 52% in ICU patients.^(4-8)^ In studies conducted on COVID-19 patients, the incidence of PIH was similar, with a rate of 21 - 22.3%.^(13,14)^ In this study, PIH was detected in 53 patients (37.6%), which is in accordance with values previously reported in the literature. We excluded ETI-related cardiac arrests. However, cardiac arrest may be due to severe and abrupt hypotension. Although ETI was administered under close monitoring and with an expert team, we excluded these patients due to a likely misdiagnosis just before the cardiac arrest. Despite our specific definition of PIH compared with other broad range definitions and the exclusion of ETI-related cardiac arrests, we believe that the incidence of PIH is high in COVID-19 patients.

In studies conducted in the ICU setting, risk factors associated with PIH included advanced age, a high severity of illness, the use of NIV before ETI, intubation for acute respiratory failure, pre-ETI shock status, having a lower MAP before ETI, the use of vasopressors before ETI, the use of neuromuscular blocker agents during ETI, history of chronic obstructive pulmonary disease, and/or renal diseases.^(4-8)^ Several putative risk factors for PIH include hypovolemia, impaired systemic vascular resistance, receipt of sedative medications, and reduced venous return from positive pressure ventilation.^(19)^ The SI (heart rate/SBP) and MSI (heart rate/MAP) are simple tools that assess the severity of hypoperfusion circumstances and are easily calculated at the bedside.^(16,17)^ Previous studies have shown that high SI measures before ETI are a predictor of PIH in the ICU setting.^(5,6,16)^ High measures of SI before ETI are also associated with ICU mortality.^(16)^ Similarly, high MSI has been found to be associated with PIH in one study^(6)^ but not in another study in the ICU.^(16)^ In this study, pre-ETI measurements that may indicate shock status, including high lactate, low SBP, low MAP, high SI, and high MSI, were associated with PIH in the univariate analysis. Currently, there is no standard cutoff value of SI predicting PIH. A study found that a pre-ETI SI value ≥ 0.90 was significantly associated with PIH.^(16)^ Similarly, in this study, a SI value ≥ 0.90 was independently associated with PIH. Some studies have examined the relationship between disease severity scores (APACHE II and SOFA) and PIH. Although higher disease severity scores were not associated with PIH in one study,^(8)^ a higher SOFA score was associated with PIH in another study.^(20)^ In this study, disease severity scores were higher in patients with PIH than in patients without PIH. Due the fact that the disease severity scores are measured on the first day of ICU admission, as well as the fact that the status of severe COVID-19 patients can change rapidly, they may not reflect the pre-ETI status. The SI may be a useful tool to predict PIH in addition to disease severity scores, such as APACHE II and SOFA.

Increased vascular permeability and acute phase responses secondary to inflammation may cause hypoalbuminemia.^(21)^ Albumin has important effects on the maintenance of intravascular colloidal osmotic pressure; therefore, hypoalbuminemia can cause fluid exchange from blood vessels to tissues.^(21,22)^ Fluid exchange may worsen intravascular volume status in patients with septic shock, which is a type of vasodilatory or distributive shock.^(23)^ In septic patients who are treated with albumin plus crystalloid, the suspension time of vasopressor or inotropic agent administration was shorter than in septic patients treated with crystalloid.^(24)^ We found that, when compared with higher levels, low albumin levels independently increased the risk of PIH. An albumin level of < 2.92g/ dL can be used to predict PIH. The association between low albumin levels and PIH may be due to the lowered intravascular colloidal pressure.

In this study, we assessed the impact of PIH on AKI, due to the fact that hypotension is a risk factor for AKI in septic ICU patients.^(25)^ In a meta-analysis, the pooled AKI incidence was 39.0% in ICU patients and 42.0% among deceased patients with COVID-19.^(26)^ In this study, the incidence of AKI was high at a rate of 64.5%, and AKI occurred in the post-ETI period in 48.2% of the patients. We found no difference between the two groups in terms of AKI development in the post-ETI period. Higher blood urea nitrogen (BUN) levels in the PIH group may be associated with a greater number of patients with AKI in this group or can possibly be related to hypovolemic volume status.

There is no proven relationship between high procalcitonin levels and PIH. In this study, patients with PIH had higher levels of procalcitonin than patients without PIH. The serum procalcitonin levels reflect the severity of the disease and are associated with poor outcomes in COVID-19 patients.^(27)^ In this study, patients with high procalcitonin levels had more PIH, likely because they had more severe disease than those with low levels.

Hospital mortality was 87.9% (n = 124) in this specific cohort of patients who underwent IMV. In a meta-analysis including 57,420 adult patients with COVID-19 who received IMV, the overall reported CFR was estimated to be 45% (95%CI 39 - 52%).^(11)^ In this meta-analysis, among studies in which age-stratified CFR was available, pooled CFR estimates were 84.4% (95%CI 83.3 - 85.4%) in patients aged above 80 years.^(11)^ Similarly, the mortality of patients who underwent IMV in a large study was 88.1%.^(12)^ In the literature, the highest mortality rate in patients who underwent IMV was 97%.^(28)^ The high mortality of our patients was attributed to advanced age, multiple complicated pre-existing comorbidities, and a vulnerable patient population undergoing IMV. Furthermore, mortality was similar in the PIH and non-PIH groups.

### Limitations and strengths of the study

The limitations of the study are as follows: we did not evaluate several other ETI-related complications, such as aspirations; in patients admitted from the emergency department and who were immediately intubated after admission to the ICU, the prehospital history of antihypertensive drug use and fluid intake data may be insufficient; and the mortality rate in this study was relatively high. The high mortality rate in both the PIH and non-PIH groups may have resulted in the inability to identify the association between PIH and adverse outcomes and mortality. The strengths of the study are as follows: ETI procedures were conducted by expert physicians; we excluded conditions such as cardiac arrest, more than two attempts for ETI, and pre-ETI hypotension that may have confounded the data; and this study was conducted on a homogeneous group with hypoxemic respiratory failure due to COVID-19.

## CONCLUSION

We found a high incidence of postintubation hypotension in critically ill COVID-19 patients. Shock index ≥ 0.90, albumin levels < 2.92g/dL, and higher procalcitonin levels compared to lower procalcitonin levels were independently associated with postintubation hypotension. The shock index can be calculated inexpensively, easily, and quickly at the bedside prior to endotracheal intubation. A shock index ≥ 0.90 may be a practical tool to estimate the increased risk of postintubation hypotension. The use of this parameter may contribute to the optimization of therapies to prevent postintubation hypotension in vulnerable patients with severe COVID-19.
